# Correlation between anti-retinal antibodies and lupus retinopathy in systemic lupus erythematosus

**DOI:** 10.1038/s41598-026-44125-z

**Published:** 2026-03-13

**Authors:** Qingyi Zou, Linqi Zhang, Muzi Li, Bohao Wang, Yu Cao, Xuewu Zhang, Jinfeng Qu

**Affiliations:** 1 Department of Ophthalmology, Peking University People’s Hospital, Beijing, China; 2 Beijing Key Laboratory of Ocular Disease and Optometry Science, Peking University People’s Hospital, Beijing, China; 3 Department of Rheumatology and Immunology, Peking University People’s Hospital, Beijing, China

**Keywords:** Systemic lupus erythematosus, Lupus retinopathy, Anti-α-enolase antibody, Anti-arrestin antibody, Anti-recoverin antibody, Anti-IRBP3 antibody, Biomarkers, Diseases, Immunology, Medical research, Rheumatology

## Abstract

Lupus retinopathy (LR) is one of the most frequent and serious ocular complications of systemic lupus erythematosus (SLE), because it may cause irreversible visual impairment. The aim of this study was to evaluate the association between serum anti-retinal antibodies levels of SLE patients and the incidence of LR. Levels of serum anti-α-enolase antibody (Ab), anti-arrestin Ab, anti-recoverin Ab and anti-IRBP3 Ab were detected in 89 SLE patients (divided into LR group and non-LR group) and 81 healthy controls by enzyme-linked immunosorbent assay (ELISA). The correlation between these four anti-retinal Ab, SLE activity and the incidence of LR was evaluated. LR group had a higher SLE disease activity index (average SLEDAI score, 18 (7) versus 9 (5), *P* < 0.001), higher frequency of pleurisy (40% versus 20.4%, *P* = 0.044) and lower level of hemoglobin (102.343 ± 23.157 versus 112.759 ± 19.678, *P* = 0.025) comparing to non-LR group. LR group had higher levels of anti-α-enolase than non-LR group (*P* = 0.033) and control group (*P* < 0.0001). The levels of anti-recoverin in LR group was higher than non-LR group (*P* = 0.036) and control group (*P* < 0.0001), while the difference was not significant between non-LR group and control group (*P* = 0.109). Using combination of anti-α-enolase Ab and anti-recoverin Ab to diagnose LR in SLE patients is more effective (with area under the receiver operating characteristic curve (AUC): 72.68%) than use anti-α-enolase Ab (AUC: 65.65%) or anti-recoverin (AUC: 61.96%) only. Our results suggested that anti-α-enolase and anti-recoverin may be used as potential biomarkers of lupus retinopathy in SLE patients.

**Supplementary Information:**

The online version contains supplementary material available at 10.1038/s41598-026-44125-z.

## Introduction

Systemic lupus erythematosus (SLE) is a chronic connective tissue disease with a wide range of clinical manifestations, characterized by production of autoantibodies directed against nuclear and cytoplasmic antigens which can affect various organs and systems^1^. Lupus retinopathy (LR) is one of the most frequent and serious ocular complications of SLE, which may cause irreversible visual impairment^2,3^. Early diagnosis of LR can be helpful to predict and prevent severe visual impairment. Therefore, development of optimal diagnostic biomarker for LR in SLE patients is highly necessary.

Previous studies have reported that anti-retinal antibodies (ARAs) are associated with autoimmune retinopathy (AIR)^4–6^, diabetic retinopathy^7^, central serous chorioretinopathy^8^, macular telangiectasia type 2^9^ and some genetic retinopathies such as retinopigmentosa, while the mechanisms of the induction of ARAs and their correlation with LR remained unknown.

Recoverin is a calcium-binding protein (molecular weight 23 kDa), which inhibits the activity of rhodopsin kinase and regulates the phosphorylation of rhodopsin. It exists in the inner segment and nucleus of photoreceptors and the outer plexiform layer of retina^10^. Anti-recoverin Ab can activated Caspase-9 and Caspase-3 dependent apoptotic pathways by inducing calcium ion influx and cause apoptosis of retinal photoreceptors^11–14^.

Alpha-enolase (ENO1) is a subtype of enolase, which has a molecular weight of 46 kDa, and catalyzed the production of ATP during glycolysis. It is widely expressed in vascular endothelial cells, cone outer segments, Müller cells and the ganglion cell layer of the retina^15^. ENO1 is closely related to inflammation, autoimmune response, tumor and mitochondria function. Anti-α-enolase plays a pathogenic role by generating immune complexes, increasing intracellular calcium ion concentration and initiating apoptosis^16,17^.

Arrestin-1 (also named S antigen) is a soluble cytoplasmic protein (molecular weight 48 kDa) which is specifically expressed in the photoreceptors. It plays an important role in signal regulation viva binding to G protein-coupled receptors, membrane receptors and a variety of intracellular soluble proteins^18^. Anti-arrestin Ab is involved in the conversion of light and dark adaptation by simple diffusion^19,20^.

IRBP3 is a sugar lipoprotein about 143 kDa, which is the main soluble protein in the photoreceptor matrix. It is secreted by photoreceptor cells, and its main physiological role is to regulate the function of cones, rods, Müller cells and retinal pigment epithelium^21^, and provide retinol and retinoic acid to the retina. In addition, the protein protects the unstable molecules from oxidation and isomerization^22^. Adequate amounts of IRBP3 are therefore essential for the structural and functional integrity of the retina, especially photoreceptor cells^23^. Currently, IRBP is considered to be involved in autoimmune uveitis and is one of the antigens of spontaneous uveitis in humans and animals^24^.

The aim of this study was to evaluate the association between these four ARAs levels and the incidence of LR in SLE patients.

## Methods

### Participants

This study enrolled 170 subjects (including 89 SLE patients and 81 healthy controls) at Peking University People’s Hospital between September 2020 and July 2022. SLE patients were further divided into 2 groups (LR group and non-LR group) according to the presence of lupus retinopathy, which was diagnosed according to fundus manifestations.

### Inclusion & exclusion criteria

The inclusion criteria were as follows: (1) age ≥ 18 years, (2) SLE patients fulfilled the revised 2009 European League Against Rheumatism (EULAR) or American College of Rheumatology (ACR) classification criteria for SLE, (3) subjects without any other eye disease or any systemic disease were enrolled as controls, (4) agree to sign informed consents.

The exclusion criteria were as follows: (1) clinical manifestations or laboratory examinations suggest a history of past or present infection or malignant tumor, (2) breastfeeding or being pregnant (3) history of taking medications that may cause fundus lesions, such as steroids or hydroxychloroquine (HCQ), (4) presence of retinal diseases other than LR, such as diabetic retinopathy or macular degeneration, (5) presence of any undetermined retinal condition, (6) unable to undertake ophthalmologic examinations, (7) subjects with high bilirubin blood samples, chylous blood samples or hemolysis, (8) subjects with coagulation disorders, (9) subjects taken immunosuppressants or biological agents.

### Data extraction

Basic information, venous blood samples and laboratory test result within one week from the blood sample collection date were recorded and SLE Disease Activity Index 2000 (SLEDAI-2 K), a global index based on the symptoms and laboratory findings was calculated according to medical records from each SLE patient. All enrolled subjects underwent systemic examinations, laboratory test and ophthalmic examinations, including best-corrected visual acuity (BCVA), intraocular pressure (IOP), fundoscopy and color fundus photography after mydriasis. All SLE patients with lupus retinopathy underwent further optical coherence tomography (OCT) and fundus fluorescein angiography (FFA) examination.

### ELISA

The serum obtained from all participants were subjected to ELISA (78687186-X, Beijing Weilaibo Biotechnology, China), according to the manufacturer’s instructions. Briefly, the calibration standards were measured at the same time as the samples. After the stop solution changed the color from blue to yellow, the intensity of the color was measured at 450 nm using a spectrophotometer. Standard curve was produced and the concentration of anti-α-enolase, anti-arrestin and anti-IRBP3 in the samples was determined respectively by comparing the OD of the samples to the standard curve.

### Statistical analysis

Sample size was calculated by using GPower 3.1. Effect size was determined by data from our previous publication^25^. The total samples size of 30 was required to achieve power of 0.8 with α set to 0.05. Statistical analyses were carried out using SPSS 26.0 and GraphPad Prism 9.5.0. The variables were tested for normal distribution using the Kolmogorov-Smirnov test. Normally distributed variables were displayed as mean ± SD (standard deviation) and compared by independent t-test (between two groups) or Scheffe test (between three groups). Non-normally distributed variables were displayed as M(IQR) (median (interquartile range)) and compared by Mann–Whitney U test (between two groups) or Kruskal-Wallis test (K-W test). When the K-W test indicated a statistically significant difference, pairwise comparisons were further conducted using Dunn test. To control the risk of false positives arising from multiple comparisons, all p-values were adjusted using the Bonferroni method. The reported p-values are the adjusted ones. Comparison of categorical variables, expressed as numbers, frequency and percentages, were done using Chi-square test. Correlations between continuous normally distributed and non-normally distributed variables were assessed by calculating Pearson correlation coefficients and Spearman’s rank correlation coefficients respectively. Receiver operating characteristic curve (ROC) and area under curve (AUC) were used to evaluate the diagnostic effect of ARAs in lupus retinopathy. The P value was two-tailed and considered as statistically significant for *P* < 0.05.

## Results

### Characteristics of participants

There are 34 SLE patients in LR group, 55 SLE patients in non-LR group and 81 patients as controls enrolled in this study. Their clinical characteristics are shown in Table 1. The median age of LR group, non-LR group and control group was 44.5(29), 34(23) and 58(47) respectively. The median age in control group was significant older than in non-LR group (*P* = 0.026). The proportion of female subjects were significant lower in control group (56/81, 69.1%) than in LR group (27/34, 79.4%) and non-LR group (49/55, 89.1%) (*P* = 0.022, by Chi-square test) (Table 1). The median SLE duration of LR group and non- LR group was 10.5 years and 6 years, ranging from 20 days to 50 years and 6 months to 42 years respectively.

**Table 1:** Characteristics of participants.

Item	LR group (*n* = 34)	non-LR group (*n* = 55)	control group (*n* = 81)	*P* value
Age/year, M(IQR)	44.5 (29)	34 (23)	58 (47)	0.031*
Age range	18ཞ73	17ཞ81	17ཞ87	
Gender, n (%)				
Male	7 (20.60)	6 (10.90)	25 (30.9)	0.022*
Female	27 (79.40)	49 (89.1)	56 (69.1)	
SLE duration				
Median (y)	10.5	6	NA	
Range	20 d ~ 50	6 m~42y	NA	

*P < 0.05; *SLE* systemic lupus erythematosus, *M* median, *IQR* interquartile range, *d* days, *m* months, *y* years.

### Clinical manifestations and laboratory examinations of SLE patients

The comparison of clinical manifestations between LR group and non-LR group is shown in Table 2. The median SLEDAI score of the LR group was 18 (IQR = 7), which is significantly higher than that of non- LR group (9[5], *P* < 0.001 by U test) (Table 2). The frequency of pleuritis is significantly higher in LR group than in non-LR group (40% versus 20.4%, *P* = 0.044 by Chi-square test) (Table 2).

**Table 2:** Clinical manifestations of SLE patients.

Item	LR group *n* = 34	Non-LR group *n* = 55	t/U/χ2	*P* value
SLEDAI score, M (IQR)	18 (7)	9 (5)	197.500	< 0.001*
Fever, n (%)	25 (71.4)	37 (68.5)	0.085	0.771
Tmax during fever (℃), mean ± SD	38.887 ± 0.693	38.943 ± 0.741	−0.288	0.774
Erythra, n (%)	13 (37.1)	26 (48.1)	1.045	0.307
Butterfly erythema, n (%)	16 (45.7)	21 (38.9)	0.407	0.523
Arthritis, n (%)	22 (62.9)	36 (66.7)	0.136	0.713
Alopecia, n (%)	18 (51.4)	20 (37.0)	1.798	0.180
Canker sore, n (%)	13 (37.1)	11 (20.4)	3.033	0.082
Photo allergy, n (%)	5 (14.3)	12 (22.2)	0.866	0.352
Raynaud phenomenon, n (%)	6 (17.1)	12 (22.2)	0.340	0.560
Urinary occult blood, n (%)	15 (42.9)	13 (24.1)	3.475	0.062
Urinary albumin, n (%)	20 (57.1)	26 (48.1)	0.688	0.407
Pleuritis, n (%)	14 (40.0)	11 (20.4)	4.051	0.044*
Pericarditis, n (%)	10 (28.6)	10 (18.5)	1.232	0.267

*P < 0.05; *SLEDAI* systemic lupus erythematosus disease activity index, *SD* standard deviation, *M* median, *IQR* interquartile range, *Tmax* maximum body temperature.

The comparison of laboratory examinations between LR group and non-LR group is shown in Table 3. All examination results showed no significant difference except hemoglobin which was lower in LR group than in non-LR group (102.343 ± 23.157 versus 112.759 ± 19.678, *P* = 0.025 by t test) (Table 3).

**Table 3:** Laboratory examinations of SLE patients.

Item	LR group *n* = 34	non-LR group *n* = 54	t/U/χ^2^	*P* value
CRP (mg/L), M (IQR)	2.100 (6.880)	1.210 (6.830)	863.500	0.491
ESR (mm/h), M (IQR)	27.500 (33.750)	18.000 (31.000)	741.500	0.130
IgA (g/L), M (IQR)	2.340 (1.820)	2.425 (1.690)	924.000	0.860
IgG (g/L), M (IQR)	13.100 (7.120)	11.800 (7.880)	785.000	0.179
IgM (g/L), M (IQR)	0.720 (0.790)	0.899 (0.860)	839.000	0.498
C3 (g/L), mean ± SD	0.638 ± 0.341	0.699 ± 0.271	−0.929	0.355
C4 (g/L), M (IQR)	0.130 (0.130)	0.160 (0.150)	765.500	0.131
WBC (10^9^/L), M (IQR)	5.900 (5.400)	5.650 (3.860)	787.500	0.186
Hb (g/L), mean ± SD	102.343 ± 23.157	112.759 ± 19.678	−2.274	0.025*
PLT (10^9^/L), mean ± SD	176.200 ± 80.658	185.704 ± 74.831	−0.568	0.572
Anti-dsDNA Ab (+), n (%)	16 (45.7)	22 (40.7)	0.215	0.643
ANA (+), n (%)	31 (88.6)	52 (96.3)	0.974	0.324
Anti-Sm Ab (+), n (%)	3 (8.6)	12 (22.2)	2.824	0.093
Anti-U1 RNP Ab (+), n (%)	11 (31.4)	19 (35.2)	0.134	0.714
Anti-SSA Ab (+), n (%)	14 (40.0)	27 (50.0)	0.855	0.355
Anti-SSB Ab (+), n (%)	2 (5.7)	4 (7.4)	< 0.001	> 0.999

*P < 0.05; *CRP *C-reactive protein, *ESR *erythrocyte sedimentation rate, *IgA *immunoglobulin A, *IgG *immunoglobulin G, *IgM *immunoglobulin M, *C3 *complement 3, *C4 *complement 4, *WBC *white blood cell count, *Hb *hemoglobin, *PLT *blood platelet count, *dsDNA *double stranded, DNA, *U1 RNP *U1 ribonucleoprotein, *Anti-SSA Ab *anti-Sjo gren syndrome A antibody, *Anti-SSB Ab *anti-Sjo gren syndrome B antibody, *SD *standard deviation, *M *median, *IQR *interquartile range.

### Fundus manifestations and retinal features of fundus group

In LR group, fundus examinations revealed various abnormalities (Table 4): retinal hemorrhage (47.1%), soft exudates (cotton-wool spots) (26.5%), retinal vasculitis (23.5%), macular edema (5.9%), retinal artery occlusion (5.9%), retinal vein occlusion (8.8%), papilledema (5.9%), optic atrophy (5.9%), retinal detachment (2.9%), vitreous hemorrhage (2.9%) and choroidal detachment (2.9%).

**Table 4:** Fundus manifestations and retinal features of LR group.

Item	Case	Proportion
Retinal hemorrhage	16	47.1%
Cotton-wool spots	9	26.5%
Retinal vasculitis	8	23.5%
Macular edema	2	5.9%
Retinal artery occlusion	2	5.9%
Retinal vein occlusion	3	8.8%
Papilledema	2	5.9%
Optic atrophy	2	5.9%
Retinal detachment	1	2.9%
Vitreous hemorrhage	1	2.9%
Choroidal detachment	1	2.9%

### Levels of ARAs

**Fig. 1:**
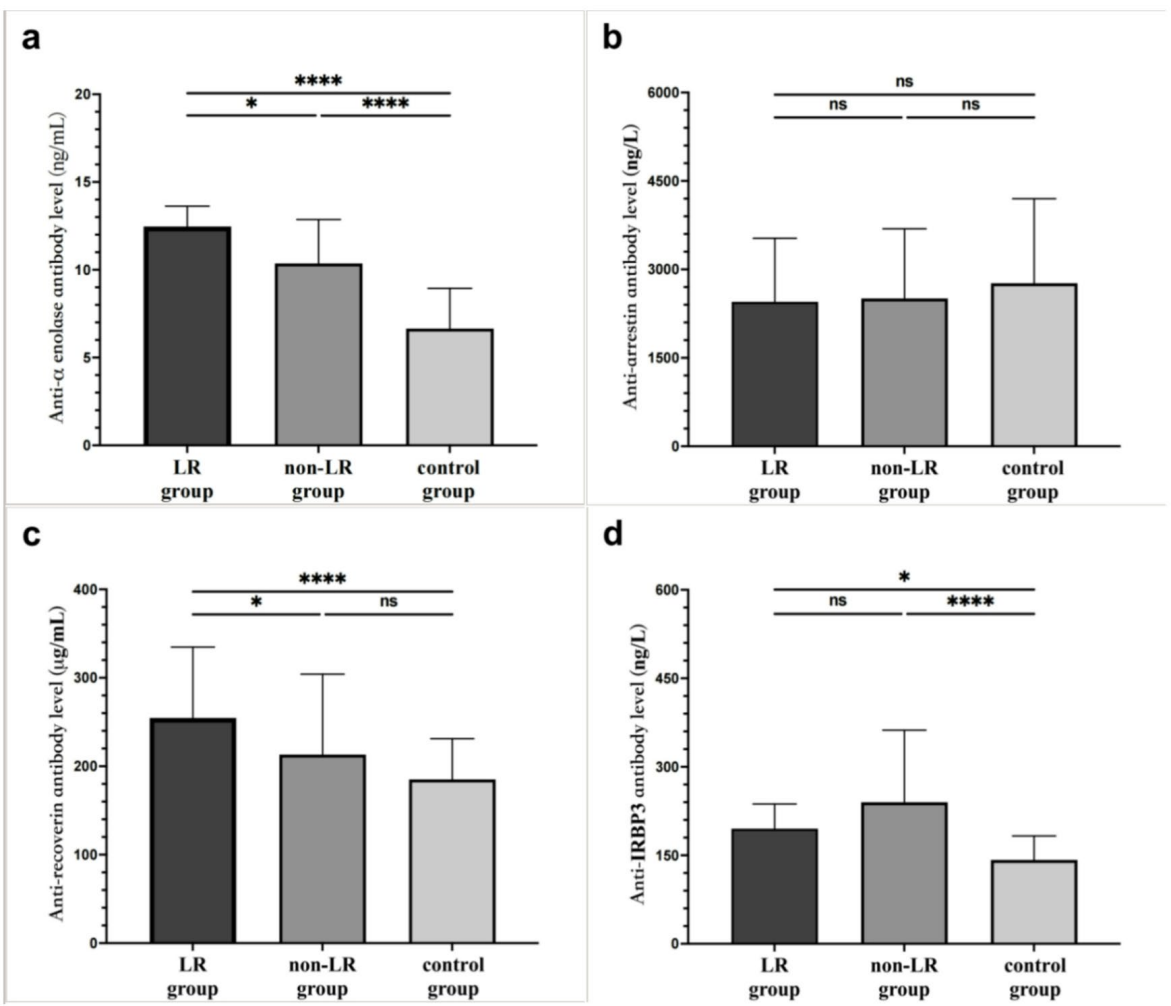
The level of ARAs in LR group, non-LR group and control group. (**** *P* < 0.0001;* *P* < 0.05;ns, not significant).

The level of four ARAs in enrolled participants is shown in Table 5; Fig. 1. K-W test showed significant difference of anti-α-enolase Ab level among the three groups (*p* < 0.001). LR group had higher level of anti-α-enolase Ab compared with non-LR group (12.47 (3.38) versus 10.36 (5.13) ng/ml, *P* = 0.033 by Dunn test) (Table 4). Additionally, both LR group and non-LR group had a significantly higher level of anti-α-enolase Ab than control group (12.47 (3.38) versus 6.65 (3.69) ng/ml, *P* < 0.0001 by Dunn test; 10.36 (5.13) versus 6.65 (3.69) ng/ml, *P* < 0.0001 by Dunn test).

K-W test showed significant difference of anti-recoverin Ab level among the three groups (*p* < 0.001). Level of anti-recoverin Ab were significantly higher in LR group than in non-LR group (254.54 ± 80.14 versus 213.22 ± 90.89 µg/mL, *P* = 0.036 by Scheffe test) and control group (254.539 ± 80.135 versus 185.150 ± 45.959 µg/mL, *P* < 0.0001 by Scheffe test) (Table 4). While there is no significant difference between non-LR group and control group (213.219 ± 90.886 versus 185.150 ± 45.959 µg/mL, *P* = 0.109 by Scheffe test).

Level of anti-arrestin Ab in three groups were 2449.66 (1795.12) ng/L in LR group, 2507.97 (1747.84) ng/L in non-LR group and 2766.20 (2014.00) ng/L in control group respectively and there was no statistical difference among these three groups (H = 5.488, *P* = 0.064 by Kruskal-Wallis test).

K-W test showed significant difference of anti-IRBP3Ab level among the three groups (*p* < 0.001). Both LR group and non-LR group had a significantly higher level of anti-IRBP3 Ab than control group (195.10 (100.19) versus 142.26 (65.15) ng/L, *P* = 0.024 by Dunn test; 239.93 (219.04) versus 142.26 (65.15) ng/L, *P* < 0.0001 by Dunn test). While level of anti-IRBP3 Ab between LR group and non-LR group showed no significant difference (195.10(100.19) versus 239.93 (219.04) ng/L, *P* = 0.838 by Dunn test).

**Table 5:** The level of ARAs of all participants.

ARAs	LR group	Non-LR group	Control group	H/F	*P* value
α-enolase Ab/ng/ml, M (IQR)	12.47 (3.38)^†‡^	10.36 (5.13) ^†#^	6.65 (3.69) ^‡#^	53.71	< 0.001*
recoverin Ab/µg/ml, mean ± SD	254.54 ± 80.14^†‡^	213.22 ± 90.89^†^	185.15 ± 45.96^‡^	11.56	< 0.001*
arrestin Ab/ng/L, M (IQR)	2449.66 (1795.12)	2507.97 (1747.84)	2766.20 (2014.00)	5.49	0.064
IRBP3 Ab/ng/L, M (IQR)	195.10 (100.19) ^‡^	239.93 (219.04) ^#^	142.26 (65.15) ^‡#^	21.20	< 0.001*

*K-W test among three groups, *P* < 0.05; ^†^Dunn test between LR and non-LR group, *P* < 0.05; ^‡^Dunn test between LR and control group, *P* < 0.05; ^#^Dunn test between non-LR and control group, *P* < 0.05; *IRBP3 *interphotoreceptor retinol binding protein 3, *SD *standard deviation, *M *median, *IQR *interquartile range.

**Fig. 2:**
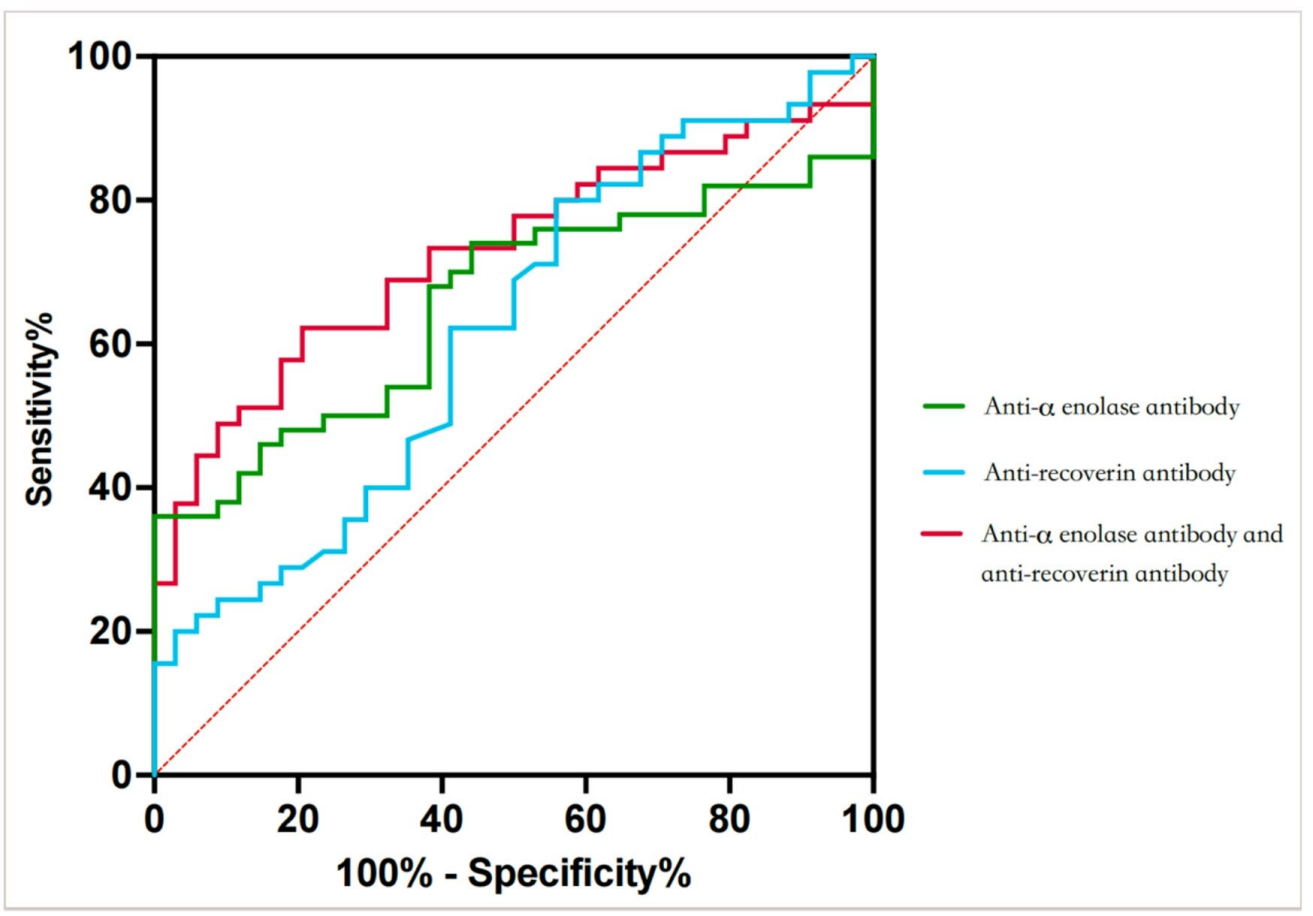
Receiver operating characteristic (ROC) curve for diagnosing LR by anti-α-enolase Ab, anti-recoverin Ab or their combination.

In order to further evaluate the diagnostic power of anti-α-enolase Ab and anti-recoverin Ab in LR, receiver operating characteristic curve (ROC) was produced according to the level of these two antibodies, alone and in combination (shown in Table 6; Fig. 2). ROC analysis was performed between the LR group and the non-LR group, the non-LR group was used as the basis. When using anti-α-enolase Ab alone, the area under curve (AUC) was 0.6565 (*P* = 0.0153). When using a cutoff value of 8.90 ng/mL, the sensitivity and specificity of anti-α-enolase Ab alone for diagnosing LR was 36.0% and 100.0%, respectively. When using anti-recoverin Ab alone, the AUC was 0.6196 (*P* = 0.0700). When using a cutoff value of 260.10 µg/mL, the sensitivity and specificity of anti-recoverin Ab alone for diagnosing LR was 80.0% and 44.1%, respectively. When using predict probability of LR calculated by using anti-α-enolase Ab combined anti-recoverin Ab binary logistic regression model, the AUC was 0.7268 (*P* = 0.0006). Using 0.3968 as a cutoff value of predict probability, the sensitivity and specificity for diagnosing LR was 62.2% and 79.4%, respectively.

**Table 6:** Summary of ROC results.

Classification	AUC	*P* Value	Cutoff value	Sensitivity/%	Specificity/%
anti-α-enolase Ab	0.6565	0.0153*	8.90	36.0	97.1
anti-recoverin Ab	0.6196	0.0700	260.10	80.0	44.1
combination of anti-α-enolase and anti-recoverin Ab	0.7268	0.0006***	0.3968	62.2	79.4

*P < 0.05; ***P < 0.001; *AUC* area under curve.

## Discussion

In this study, the incidence of LR was 38% (34/89) which was substantially higher than that reported in some prior studies. This discrepancy may be attributed to selection bias. Since the SLE patients included in our cohort were all hospitalized, they likely exhibited more severe SLE conditions compared to non-hospitalized populations, which could consequently lead to a higher observed incidence of LR. Therefore, caution should be exercised when extrapolating the findings of this study to larger populations due to the presence of this selection bias. In our results, we reported the average SLEDAI score of the enrolled patients, which, to some extent, allows readers to gauge the disease activity level of this specific cohort and helps mitigate the impact of selection bias on the interpretation of the results.

In this study, the fundus manifestations of the LR group were diverse, including retinal hemorrhage, cotton-wool spots, retinal vasculitis, macular edema, retinal artery occlusion, retinal vein occlusion, papilledema, optic atrophy, retinal detachment, vitreous hemorrhage and choroidal detachment. The most common fundus abnormalities in our enrolled patients were retinal hemorrhage (47.1%), cotton-wool spots (26.5%) and retinal vasculitis (23.5%). This result was consistent with previous reports, indicating good representative features of LR group enrolled in this study. However, due to the lack of a recognized grading scale for LR and the diversity of manifestations among LR in different phase, we did not analyze the correlation between the severity of fundus abnormalities and hematological indicators in this study. We consider to score fundus manifestations more specifically and further analyze the relationship between the severity of fundus lesions and the severity of systemic lesions and serological markers in future studies.

LR group had a higher SLEDAI score than non-LR group, suggesting that patients with LR had higher disease activity, which was consistent with previous studies. However, it should be noted that in recent years, SLEDAI have included LR into the scoring rules, assigning a score of 8 points. If we subtracted these 8 points and then compared the left SLEDAI score of LR and non-LR, the difference between two groups was not statistically significant (U = 831.500, *P* = 0.380), suggesting that there may not be a definite association between LR and other general systemic lesion in SLE, this finding was similar to what Bashiri et al. reported in 2021^26^. However, our study found that LR group had higher frequency of pleurisy and lower level of hemoglobin, suggesting that there may be a link between LR and some specific systemic manifestations, which needs larger sample sizes investigation to confirm.

Our previous study has found that the serum level of anti-recoverin Ab increased in SLE patients with LR^25^, however, due to the lack of data on specific manifestations of fundus lesions in previous studies, we could not know whether there was selective bias. In this study, we found that the level of anti-recoverin Ab was significantly higher in LR group than in non-LR group and control group, and no difference between the latter two groups was observed, which further confirmed our previous studies.

Previous publications report anti-α-enolase Ab was observed positive in 27.14%(19/70) of autoimmune diseases patients and 45.8%(11/24) of SLE patients^27^, and the serum level of anti-α-enolase Ab in the disease group was significantly higher than the healthy group^28^. Huang et al. suggested that anti-α enolase Ab combined with urinary β2 microglobulin can predict the incidence of nephritis in SLE patients with a sensitivity of 92% and specificity of 93%^29,30^. In this study, the serum anti-α-enolase Ab level in LR group was elevated compared with non-LR group and the AUC was 0.6565 when predicting LR by this antibody alone. No correlation was found between LR and kidney involvement biomarker such as urinary occulted blood and urinary albumin. Considering the structural similarity between retina and glomerulus, the level of urinary β2 microglobulin can be investigated in future studies to observe whether there is a certain correlation between its level and LR.

The difference in serum anti-arrestin Ab levels among the three groups was not statistically significant and there was no statistically significant difference in the level of anti-IRBP3 Ab between LR group and non-LR group in this study. One possible explanation may be because these two Ab were only expressed in the retinal photoreceptor and the damage of photoreceptor cells often occurs secondary to deep retinal ischemia and hypoxia and may not be affected in all patient especially in the early stage. Optical coherence tomography (OCT) could be a valuable tool for the assessment of photoreceptor damage, but unfortunately the OCT images of most of our participants were lacking.

Our study has many limitations. Firstly, the sample size is small, especially in the LR group and it is difficult to avoid selection bias during the analysis of results. Secondly, the control group does not fully match the SLE groups in terms of age and gender. This may create a potential confounding variable. Third, some possible biomarkers which may be associated with LR were not included in our study, such as urinary β2-microglobulin, as we mentioned above. Thirdly, this study only analyzed ARAs in blood sample and their level in aquas humor might be different to its serum. Further studies are needed to provide more solid data on the level of ARAs in aqueous humor and exploring their association between them and LR.

In conclusion, this study is the first to evaluate the association between serum anti-retinal antibodies levels of SLE patients and the incidence of lupus retinopathy. We found SLE patients with LR had higher frequency of pleurisy and lower level of hemoglobin compared with SLE patients without LR. Anti-α-enolase Ab and anti-recoverin Ab may be used as potential biomarkers of lupus retinopathy in SLE patients, and the combined diagnostic effect is better than these two antibodies alone.

## Supplementary Information

Below is the link to the electronic supplementary material.

Supplementary Material 1

## Data Availability

All data generated or analysed during this study are included in this published article (and its Supplementary Information files).
